# Understanding the Mosaic of COVID-19: A Review of the Ongoing Crisis

**DOI:** 10.7759/cureus.7366

**Published:** 2020-03-22

**Authors:** Madeeha Subhan Waleed, Waleed Sadiq, Muneeba Azmat

**Affiliations:** 1 Internal Medicine, Capital Hospital, Islamabad, PAK; 2 Internal Medicine, Staten Island University Hospital, New York, USA; 3 Internal Medicine, Shaukat Khanum Cancer Memorial Hospital, Lahore, PAK

**Keywords:** incubation, pneumonia, corona virus, covid-19, wuhan coronavirus, pandemic, coronavirus

## Abstract

In late 2019, a queer type of pneumonia emerged in Wuhan city in the central part of China. On investigation, it was found to be caused by the coronavirus. Human coronaviruses were discovered in the 1960s. There are a total of seven types of coronaviruses that infect humans: 229E and NL63 are the alpha coronaviruses; OC43, HKU1, MERS-CoV, and SARS-CoV are beta coronaviruses, and SARS-CoV-2 or COVID-19 is a novel coronavirus. COVID-19 surfaced in China at the culmination of the year 2019. The pandemic then fanned out rapidly, involving Italy, Japan, South Korea, Iran, and the rest of the world.

## Introduction and background

**Figure 1:**
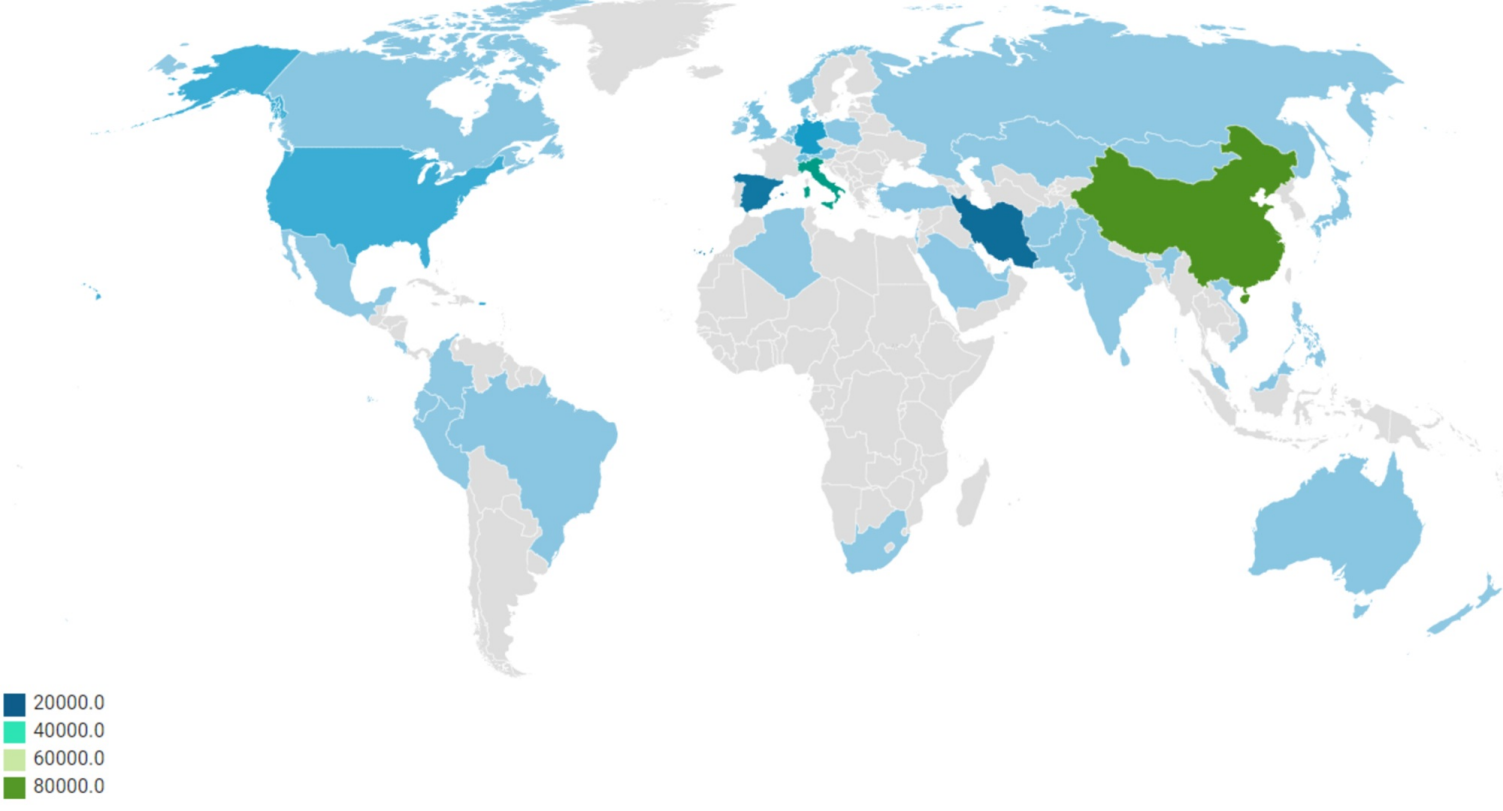
Prevalence of COVID-19 cases around the world

Six Coronavirus species are culprits of human ailments [1]. The emergence of the severe acute respiratory syndrome (SARS) in 2002-2003 and the Middle East respiratory syndrome (MERS) in 2012 showed the transferal of virus between animal‐to‐human and human‐to‐human [2-3]. In December 2019, a new virus named COVID-19 surfaced in Wuhan [4]. It has rapidly disseminated across China and several other countries [5-11]. Initial cases were reported in December 2019 [12]. These cases of mysterious pneumonia in Wuhan were communicated to the World Health Organization (WHO) on the last day of 2019 [13]. Later, on January 7, 2020, Chinese health authorities asserted that this was due to a novel coronavirus, 2019-nCoV [14]. On January 30, 2020, 9976 cases were revealed in at least 21 countries [15]. The blaze has since then escalated, involving the whole of China and 27 other countries, raising the number of cases to 70,000 on Feb 17, 2020 [16]. In March, it was reported that COVID-19 has dispersed in Europe. China, on the other hand, enforced robust measures to fight against COVID-19 by social distancing, early detection of the cases, immense lazaretto, and seclusion of infected individuals. All these measures helped flatten the curve of spread in China, Hong Kong, and Singapore. South Korea used digital technology to trace the contacts thus containing the virus without the lockdown. According to the John Hopkins University case dashboard, the prevalence cases as of March 19, 2020, mounted to 81,155 in China alone (Figure 1).

## Review

Symptoms

The COVID-19 infection has an incubation period varying between two and 14 days, with an average of roughly five days [17]. The spectrum of disease manifestation is variable, thus complicating the diagnosis of the infected. The majority of patients with COVID-19 are either asymptomatic or have mild symptoms consistent with upper respiratory tract infection. The most typical symptoms of COVID-19 illness are fever, dry cough, and malaise. Other symptoms include headache, productive cough, shortness of breath, hemoptysis, diarrhea, and decreased lymphocytes on complete blood count [18-20]. A study on the spread of SARS‐CoV in health care workers showed 7.5% were SARS-positive cases with no symptoms at all. They were linked with a smaller number of SARS antibody titers and the higher use of face masks [21]. Another study revealing a toddler with no symptoms of the disease but still carrying the COVID-19 virus highly suggested that asymptomatic patients are active carriers of the infections [22].A study showed that 33 (52%) of 64 patients were interrogated, with 26 (79%) of them confirming at least one respiratory symptom [23]. Serious symptoms, such as respiratory failure, septic shock, and multiple system involvement, were present in 5% of the cases [24].

Transmission

COVID-19 owns strong pathogenicity and transmissibility, even more than the other members of the family (SARS‐CoV and MERS‐CoV) [25]. Wrapp et al. stated that COVID-19 binds to angiotensin-converting enzyme 2 (ACE2) receptors [26-28]. Another hypothesis put forward by Zou L et al. states that the shedding pattern of viral nucleic acid in patients infected with COVID-19 is homogenous to influenza [29]. Other modes of transmission are being studied as COVID-19 can be detected in the gastrointestinal tract, saliva, and urine, comparing it to the influenza virus, which has the same mode of transmission [30]. Despite this, to the present, there has been no definitive evidence of coronavirus reservoirs other than mammals and birds [31-32]. The genomic sequence analysis of COVID-19 identified 88% similarity with two bat-derived SARS-like coronaviruses [33-34]. Person-to-person transmission is the most likely mode of transmission of COVID-19 infection, as some cases were detected in people who never visited the wet market of Wuhan but were residents of the area or visited the city and still contracted the virus [35]. The virus is highly transmissible, with viral shedding from nasopharyngeal aspirates for at least 24 days after the first presentation of symptoms, which is longer than the one previously reported in China [36]. The virus is highly resistant, staying on surfaces for days and in the air for hours. Lancet also reminded doctors not to ignore that COVID-19 can also spread through the ocular medium, as the infected droplets might contaminate conjunctiva and cause infection [31]. A single infected person can spread the disease to at least two to three other individuals. The drastically increasing number of cases points towards more of a human-to-human spread of the disease.

Treatment options

Currently, COVID-19 infection has no treatment or vaccine available for the potential cure of humans. Antiviral drugs, such as nucleoside analogs and human immunodeficiency virus (HIV) protease inhibitors can be used to weaken the virus until a particular drug becomes available [37]. Clinical studies state that Remdesivir (GS5734) (that hinders ribonucleic acid (RNA) polymerase) could be used against coronavirus infections [38]. Another study states the importance of chloroquine in reducing the morbidity of COVID-19 pneumonia [39-40]. Another study tried treating 75 patients twice a day with the oral administration of 75 mg oseltamivir, 500 mg lopinavir, 500 mg ritonavir, and intravenous (IV) 0.25 g ganciclovir for three to 14 days [41]. A randomized control trial was conducted in Wuhan, China, to determine the effectiveness of lopinavir-ritonavir against COVID-19 but the results were pretty discouraging [42]. A phase 1 trial for an investigational vaccine has also begun in Seattle. Multiple therapeutic treatments are still under investigation and further trials are necessary in this time of distress.

Future prognosis

There is currently no information available regarding virus mutation [43]. The current time requires studying the virus mutations with respect to their geography and pliability to human hosts [44]. Children may suffer from mild disease. Fatality is high in elderly people with accompanying chronic underlying diseases [45]. To reduce the havoc caused by COVID-19, public health and infection control should take actions drastically [46]. Travel history should highly be taken into account for the early detection and isolation of COVID-19 cases [47]. Every measure should be taken to reduce the progression of the malady until health care authorities find appropriate therapeutics and vaccines [48]. China shut down the whole country and Italy is currently walking down the same path. People in the United States and from around the world have decided to self-quarantine. A travel ban from highly affected countries has been administered. Flattening the curve amidst the chaos is vital. Testing for the virus should be readily available. Further studies should be conducted studying the virus course. A vaccine against the virus should be made as soon as possible. All the countries should make a conjoint effort, crossing borders in inventing the specific antiviral and vaccine against the evil COVID-19.

## Conclusions

The last pandemic to hit the world before COVID-19 was the H1N1 pandemic of 2009, which claimed almost 12,469 lives in the United States alone. COVID-19 is a similar pandemic and specific measures should be administered to halt the spread of the disease, especially taking into account the high-risk population that includes children, health care professionals, and the elderly population. Social distancing, avoiding gatherings, wearing a mask, and washing hands with soap and water can help halt the spread to some extent. Strong, multifaceted action should be embarked against COVID-19 so that the deceleration phase of the disease begins. The Chinese approach to the disease has worked and all countries should have a similar approach by limiting social gatherings, shutting down schools and malls and urging people to work from home. Countries should allow adequate testing, the isolation of people with COVID-19, contact tracing, and forcing quarantine of all contacts. Since asymptomatic patients can spread the disease, its transmission should be studied in much more detail.
